# Awareness, Framework-Based Proficiency, and Clinical Implementation of Ankle Foot Orthosis–Footwear Combination (AFO–FC) Tuning: A Cross-Sectional Survey

**DOI:** 10.3390/jcm15082846

**Published:** 2026-04-09

**Authors:** Amneh Alshawabka, Wa’el Qa’dan, Mahmoud Alfatafta, Huthaifa Atallah, Anthony McGarry, Bálint Molics

**Affiliations:** 1 Department of Prosthetics and Orthotics, School of Rehabilitation Sciences, The University of Jordan, Amman 11942, Jordan; hzvhh3@tr.pte.hu (W.Q.); m.alfatafta@ju.edu.jo (M.A.); h.atallah@ju.edu.jo (H.A.); 2 3D Printing and Visualisation Centre, University of Pécs, 7624 Pécs, Hungary; 3 Biomedical Engineering Department, University of Strathclyde, Glasgow G4 0NW, UK; anthony.mcgarry@strath.ac.uk; 4 Department of Sport Physiotherapy, Faculty of Health Sciences, University of Pécs, 7621 Pécs, Hungary; molics.balint@etk.pte.hu

**Keywords:** ankle foot orthosis, AFO–footwear combination, orthotic tuning, shank–to–vertical angle, awareness, clinical implementation, framework-based proficiency, rehabilitation

## Abstract

**Background:** Ankle foot orthosis–footwear combination (AFO–FC) tuning involves structured adjustment of the AFO relative to footwear to optimise shank alignment and ground reaction force (GRF) positioning during stance. Although established biomechanical frameworks and clinical algorithms are available, variability in clinical implementation persists. Previous investigations have primarily relied on self-reported practice within single healthcare settings and have not, to our knowledge, systematically examined how orthotists articulate and apply tuning principles within structured clinical reasoning across diverse educational and practice environments. **Objectives:** This study aimed to determine the level of awareness and framework-based proficiency in AFO–FC tuning among practising orthotists in a geographically diverse convenience sample, to examine the extent to which AFO–FC tuning is integrated into routine clinical practice, and to explore associations between framework-based proficiency level and selected professional characteristics. **Methods:** A cross-sectional study was conducted using an online survey of practising orthotists (*n* = 245). Awareness of AFO–FC tuning and self-reported routine implementation were assessed. Framework-based proficiency was evaluated among respondents reporting awareness (*n* = 212) using structured content analysis of open-text responses within a predefined exploratory five-domain biomechanical framework, and classified as limited (0–1 domains), partial (2–3 domains), or full (4–5 domains). Associations between framework-based proficiency level and professional characteristics were examined using chi-square tests. Binary logistic regression was performed to assess the association between framework-based proficiency level and self-reported routine implementation. **Results:** Self-reported awareness of AFO–FC tuning was high (86.5%), whereas 53.5% reported routine implementation. Based on the framework scoring, 59.0% demonstrated limited framework-based proficiency, 31.6% partial framework-based proficiency, and 9.4% full framework-based proficiency. No statistically significant associations were observed in this sample between framework-based proficiency level and educational qualification, years of clinical experience, or annual AFO case volume (*p* > 0.05). Full framework-based proficiency was associated with higher odds of self-reported routine implementation (OR = 4.03, 95% CI 1.44–11.25, *p* = 0.008). **Conclusions:** Despite high self-reported awareness, framework-based proficiency in AFO–FC tuning was limited within this sample. Self-reported routine implementation was more frequently reported among respondents with higher framework-based proficiency, whereas no statistically significant associations were observed with educational level, clinical experience, or annual AFO case volume. These hypothesis-generating findings should be interpreted cautiously given the cross-sectional design and framework-based (non-validated) classification.

## 1. Introduction

Ankle foot orthoses (AFOs) are external devices designed to encompass the ankle joint and all or part of the foot in order to control motion, provide stability and improve functional gait [1]. A wide range of AFO designs is available, differing in trimline configuration, material properties, and structural stiffness [2,3]. These design characteristics determine how forces are transmitted to the limb and therefore influence the mechanical behaviour and clinical effectiveness of the orthosis [4].

Rigid AFOs represent a non-articulated configuration intended to restrict motion between the shank and the foot [1]. They are frequently prescribed for individuals with equinovarus deformity, where control of excessive plantarflexion and mediolateral instability is required [5,6]. By restricting excessive plantarflexion and controlling sagittal ankle position, rigid AFOs control shank (tibial) progression during stance and indirectly affect the alignment of the ground reaction force (GRF) relative to the knee and hip joints [4,7]. However, if the AFO–footwear configuration does not permit adequate forward shank inclination, tibial progression during mid-stance may be restricted, potentially compromising stance stability and reinforcing abnormal joint moments [6,8]. Thus, both the mechanical characteristics of the AFO and its effects on tibial progression are critical to achieving optimal functional outcomes.

The clinical effectiveness of a rigid AFO depends on how its mechanical properties, including stiffness, alignment, and interaction with footwear, are configured to meet patient-specific functional needs. To achieve optimal outcomes, stiffness and alignment should be optimised through tuning: AFOs must be sufficiently stiff to provide structural support; however, excessive stiffness may unnecessarily restrict functional ankle motion, limit the ability to adapt to balance perturbations, and increase mechanical demands on proximal joints, potentially contributing to fatigue or secondary complications [9,10,11].

To maximise the biomechanical benefits of a rigid AFO, tuning is widely considered an integral component of prescription [8,12,13]. Tuning involves fine adjustment of the AFO relative to the footwear (AFO–footwear combination (AFO–FC)) to control tibial alignment and optimise GRF positioning relative to the lower limb joints during stance [6,8,12]. This process requires appropriate configuration of the ankle angle of the AFO (AA-AFO) according to available gastrocnemius length to avoid restricting knee extension. Owen proposed a hierarchical ankle range framework comprising three levels: R1a, R2, and R3 [8]. R1a represents the maximum passive ankle dorsiflexion measured with the knee fully extended and reflects gastrocnemius length. R2 denotes the dorsiflexion achieved within the orthosis under weight-bearing conditions, indicating the effective ankle position permitted by the AFO. R3 corresponds to the functional dorsiflexion requirement during gait, particularly during mid-stance when controlled tibial progression is essential. Together, these levels link passive musculoskeletal capacity, orthotic positioning under load, and dynamic gait demands, providing a biomechanical rationale for AA-AFO prescription.

In addition to AA-AFO, increasing attention has been directed toward the shank-to-vertical angle (SVA), the most widely used measure of shank alignment in clinical and research practice [14]. SVA is defined as the angle between the long axis of the shank and a vertical reference line and may be described as inclined, reclined, or vertical [13]. During mid-stance, a forward inclination of approximately 8–12° is generally regarded as biomechanically optimal, as it positions the knee joint centre over the midfoot and facilitates forward tibial progression [5,13]. This configuration promotes appropriate alignment of the thigh and pelvis, thereby contributing to improved stance stability [4]. SVA may exert a greater influence on gait mechanics than AA-AFO alone, and even small adjustments in shank alignment can substantially modify the biomechanical effects of a rigid AFO [13,14,15].

SVA is adjusted through tuning of the AFO–FC, enabling optimisation of GRF positioning relative to the lower limb joints. Controlled modification of heel height, wedge configuration, and footwear geometry facilitates this process [4,5,6,8,16]. Adequate forward tibial inclination facilitates appropriate GRF positioning relative to the knee and hip joint centres, thereby reducing abnormal joint moments, limiting knee hyperextension, reducing excessive hip flexion during stance, and improving spatio-temporal gait parameters such as walking speed [4,5,8,14,15,17,18,19,20,21].

Evidence indicates that AFO stiffness and alignment can modify lower-limb kinematics, energy expenditure, and functional performance during gait, with different stiffness–alignment configurations eliciting distinct biomechanical responses. In addition, variability in AFO design, including differences in material properties, stiffness, trimlines, and structural configuration, has been shown to influence biomechanical and functional outcomes [22,23,24,25]. Emerging developments in patient-specific AFOs and advanced fabrication approaches further highlight the role of design-related considerations in orthotic decision-making. Taken together, these findings emphasise the need for patient-specific optimisation of AFO design and alignment in clinical practice [9,11,16,25,26,27].

However, despite these advances, descriptions of AFO–FC alignment parameters and tuning procedures remain inconsistent in both clinical reporting and research literature. A recent scoping review [28] highlights substantial gaps in the reporting of key alignment parameters, including AA-AFO and SVA, as well as whether these parameters are individualised and optimised through tuning. Earlier work by Ridgewell et al. [29] similarly identified considerable variability and insufficient methodological detail in orthotic studies, limiting the evaluation and synthesis of evidence.

Clinical practice surveys indicate that AFO provision and clinical decision-making remain inconsistently reported and only partially aligned with published recommendations. A national survey of Norwegian orthotists working with children with cerebral palsy demonstrated that AFO provision is highly collaborative and that SVA is frequently evaluated, with instrumented gait analysis used in a subset of cases [30]. Nevertheless, discrepancies were identified between preferred AFO designs and guideline recommendations, particularly for gait patterns such as crouch gait and in children with short gastrocnemius [30]. These findings underscore the need to systematically characterise contemporary orthotic practice and to further examine how tuning-related principles are interpreted and implemented across diverse clinical contexts.

Collectively, these findings suggest that, despite the availability of established AFO–FC tuning frameworks and supporting empirical evidence [4,5,8,14,15,16,17,18,19], the reporting and application of AFO–FC tuning principles remain inconsistent across clinical and research contexts. This inconsistency may reflect the complexity of biomechanical decision-making, variability in training and clinical frameworks, and differences in access to clinical resources, including gait analysis tools.

Despite growing interest in AFO–FC tuning, implementation in routine clinical practice remains inconsistent [28,31]. These factors may limit the translation of theoretical knowledge into structured clinical application and support the need to examine both awareness and applied proficiency in current practice.

A cross-sectional survey conducted in the United Kingdom by Eddison et al. [31] reported that only approximately half of orthotists routinely incorporated AFO–FC tuning into standard practice. However, that investigation was limited to a single healthcare setting and relied primarily on self-reported practices, without independent assessment of whether participants could accurately apply biomechanical tuning principles in clinical reasoning.

To our knowledge, no study involving a geographically diverse sample has systematically evaluated orthotists’ ability to apply AFO–FC tuning principles within clinical reasoning across diverse educational and clinical practice environments. Given variation in orthotic training standards, regulatory frameworks, and healthcare infrastructure, it remains unclear whether previously reported gaps in implementation are specific to certain healthcare settings or represent a broader issue.

In the context of this study, awareness refers to whether orthotists are familiar with the concept of AFO–footwear combination (AFO–FC) tuning. Understanding reflects self-reported comprehension of the principles underlying tuning, whereas framework-based proficiency represents the ability to apply these principles within clinical reasoning, as reflected through structured coding of participants’ open-text responses within a predefined evaluation framework. Implementation refers to the self-reported routine integration of AFO–FC tuning into clinical practice. Distinguishing between these constructs is important, as they represent distinct levels of engagement with AFO–FC tuning and may not necessarily correspond to one another in clinical practice.

Accordingly, the primary aim of this study was to determine the level of awareness and framework-based proficiency in AFO–FC tuning principles among orthotists from a geographically diverse sample, evaluated using a predefined biomechanical framework grounded in established tuning standards. Secondary aims were to examine the extent to which AFO–FC tuning is incorporated into routine clinical practice, to explore associations between framework-based proficiency level and selected professional characteristics (educational qualification, years of clinical experience, and annual AFO case volume), and to identify perceived barriers to implementing AFO–FC tuning as part of routine orthotic management.

## 2. Materials and Methods

### 2.1. Study Design

A cross-sectional online survey was conducted to investigate awareness, framework-based proficiency, and clinical implementation of AFO–FC tuning among practising orthotists.

### 2.2. Ethical Approval

Ethical approval was obtained from the Institutional Review Board of the University of Jordan (IRB–JU 549/2023; approved 15 October 2023). The study was conducted in accordance with the Declaration of Helsinki [32], and electronic informed consent was obtained prior to survey access.

### 2.3. Participants and Recruitment

Eligible participants were practising orthotists actively involved in AFO prescription and/or fabrication. Responses from non-orthotists or those who did not confirm eligibility were excluded.

Participants were recruited using a non-probability sampling approach combining convenience and snowball sampling. Recruitment occurred through professional networks and social media platforms, including LinkedIn, ResearchGate, X (Twitter), Instagram, and Facebook. Profession–specific keywords in multiple languages (e.g., “orthotist,” “orthésiste,” “ortotista”) were used to broaden geographic reach. Survey invitations were sent to individuals whose publicly available profiles identified them as practising orthotists, and the survey link was additionally shared within online professional groups. Participants were encouraged to forward the link to eligible colleagues to facilitate snowball recruitment; therefore, the response rate should be interpreted as approximate.

Participation was voluntary, and recruitment through professional networks and online platforms may have introduced selection bias, as clinicians with a particular interest in AFO–FC tuning may have been more likely to respond.

Accordingly, the sample should be interpreted as a geographically diverse convenience sample rather than a representative population.

### 2.4. Data Collection

The questionnaire was administered electronically via Google Forms (Google LLC, Mountain View, CA, USA; web-based application) and was available exclusively in English to ensure consistent interpretation of biomechanical terminology and alignment with the original instrument. The English-only format may have influenced the depth and clarity of open-text responses, particularly among non-native English speakers.

Participation was voluntary and anonymous; no identifiable personal data were collected, and no incentives were provided. A reminder message was sent two months after the initial invitation to enhance participation.

Approximately 500 orthotists were contacted directly, resulting in 278 responses. Of these, 245 met the eligibility criteria and were included in the final analysis. Country of practice was recorded at the individual country level and analysed descriptively. Countries were not grouped into broader geographic regions, as regulatory frameworks, training standards, and prescribing practices may vary substantially within regions. Reporting at the country level was therefore considered more appropriate to reflect differences in national healthcare systems and professional practice structures.

### 2.5. Questionnaire Development and Adaptation

The questionnaire was adapted from the instrument developed by Eddison et al. [31], which was designed to evaluate awareness and clinical use of AFO–FC tuning among orthotists in the United Kingdom. Formal permission to adapt and use the questionnaire was obtained from the original authors prior to study initiation. Core items assessing awareness, understanding, and implementation of AFO–FC tuning were retained in their original wording to preserve comparability with previous research.

Additional items were incorporated to capture demographic characteristics, professional background, and detailed clinical reasoning related to AFO–FC tuning. Open–text questions were included to explore how respondents determine ankle angle, assess alignment, consider footwear characteristics and anticipate proximal biomechanical effects. These questions were structured in a non–directive manner and did not include specific biomechanical prompts, allowing respondents to describe their clinical reasoning in their own terms.

The additional items developed for this study were first reviewed by five orthotists with academic and clinical expertise in AFO prescription to assess clarity, relevance, and coverage of key concepts. Minor refinements were made following their feedback. The revised questionnaire was then pilot tested with 10 orthotists from the target population, recruited through professional contacts, to evaluate item clarity, comprehensibility, feasibility, and completion time. Feedback from the pilot indicated that the questionnaire was clear and practical to complete, and only minor adjustments were required prior to final administration.

The final questionnaire comprised three sections: (1) demographic and professional characteristics, including country of practice, highest educational qualification, years of clinical experience as an orthotist, and annual number of AFO cases managed, as well as role within the AFO clinical pathway and type of employing institution; (2) awareness and clinical implementation of AFO–FC tuning, adapted from Eddison et al. [31]; and (3) additional items exploring clinical reasoning related to AFO–FC tuning among respondents who reported awareness.

The questionnaire was designed to support an exploratory assessment of clinical reasoning rather than formal psychometric measurement; therefore, a full validation process (e.g., construct validity testing) was not undertaken.

The full questionnaire, including the open-text items used to assess framework-based proficiency, is provided in Supplementary Materials S1.

### 2.6. Framework-Based Proficiency Classification

**Figure 1:**
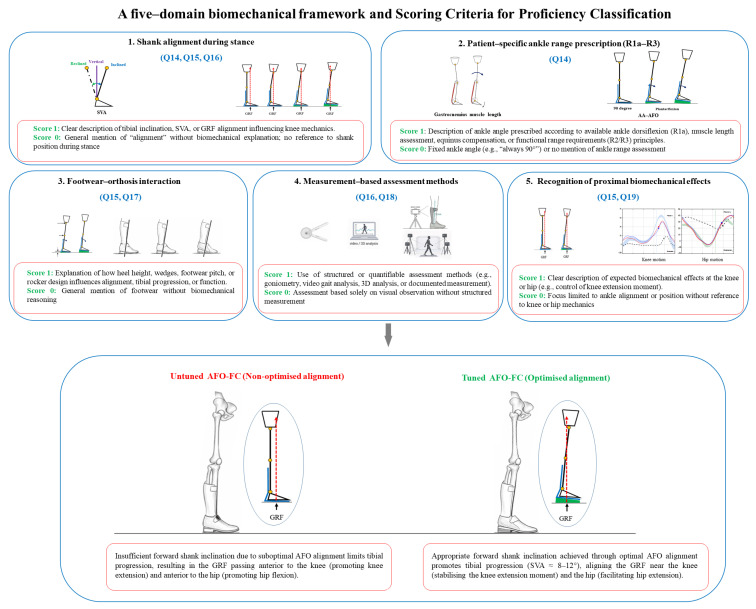
Five-domain biomechanical framework for AFO–FC tuning and illustration of untuned versus tuned alignment. The upper panels summarise the five biomechanical domains used for framework-based proficiency classification. The lower panels illustrate the difference between untuned and tuned alignment in relation to shank orientation and GRF positioning. Blue arrows indicate the adjustment of the SVA toward a more optimal forward inclination, whereas red arrows indicate the location of the GRF vector relative to the lower limb joints.

Framework-based proficiency was evaluated only among respondents who reported awareness of AFO–FC tuning (*n* = 212) through structured content analysis of open-text responses in Section 3 of the questionnaire. A five–domain biomechanical framework, informed by Owen’s hierarchical ankle range framework (R1a–R3) for AA–AFO prescription [8] and ISPO consensus recommendations [6,12] was used to guide coding. Each domain was coded as present (1) or absent (0) according to the definitions provided in Figure 1.

The open-text items were designed to elicit key components of biomechanical reasoning aligned with the predefined five-domain framework, with each item corresponding to one or more domains to support structured coding.

The five-domain framework was developed to reflect core biomechanical constructs required for AFO–FC tuning, based on established clinical principles and published literature. The selected domains—shank alignment during stance, patient-specific ankle range prescription (R1a–R3), footwear–orthosis interaction, measurement-based assessment, and proximal biomechanical effects—represent key components commonly considered in structured tuning approaches. Each domain was assigned one point, and all domains were weighted equally to capture the breadth of biomechanical reasoning rather than prioritise specific elements.

Framework-based proficiency levels were defined a priori to reflect increasing levels of demonstrated reasoning: scores of 0–1 were classified as limited proficiency, 2–3 as partial proficiency, and 4–5 as full proficiency. These thresholds reflect the number of biomechanical concepts demonstrated rather than a formally validated measure of clinical proficiency.

This approach was selected to capture the presence of key biomechanical reasoning components within participants’ responses rather than the depth or completeness of explanation, thereby supporting a structured and reproducible evaluation across respondents.

The five domain scores were added to produce a total score ranging from 0 to 5. Total scores of 4–5 indicated full proficiency, 2–3 indicated partial proficiency, and 0–1 indicated limited or no proficiency.

Clear scoring rules for each domain were established a priori to ensure consistent and objective evaluation. Prior to full coding, the two orthotists who performed the coding completed a calibration exercise using a subset of responses selected to represent a range of answer types and levels of detail, ensuring consistent interpretation of the coding framework and scoring criteria. The open-text responses were then coded independently by the same two orthotists, who were blinded to participants’ demographic and professional background information. Inter-rater agreement was calculated at the domain level across all coded judgments, and Cohen’s kappa was used to quantify agreement prior to consensus resolution of discrepancies (κ = 0.85; 95% CI 0.79–0.91). Discrepancies were resolved through discussion until consensus was reached.

### 2.7. Statistical Analysis

Data were managed in Microsoft Excel 2016 (Microsoft Corp., Redmond, WA, USA) and analysed using IBM SPSS Statistics (Version 29.0; IBM Corp., Armonk, NY, USA). Categorical variables were summarised as frequencies and percentages. 95% confidence intervals (CIs) for proportions were calculated using the Wilson score method.

Associations between framework-based proficiency level (limited, partial, full) and selected professional characteristics (educational qualification, years of clinical experience, and annual number of AFO cases managed) were examined using chi–square tests of independence. Effect sizes were quantified using Cramér’s V.

The association between framework-based proficiency level and routine implementation of AFO–FC tuning was also examined using chi–square analysis. Binary logistic regression was subsequently performed to assess the association between framework-based proficiency level and routine implementation (yes/no). Limited framework-based proficiency level was used as the reference category. Odds ratios (ORs) with 95% CIs were reported.

All statistical tests were two–tailed, and statistical significance was set at *p* < 0.05.

Chi-square test assumptions were assessed by examining expected cell counts in each contingency table. Where small expected frequencies were identified, results were interpreted with caution, and effect sizes (Cramér’s V) were reported to support interpretation. Given the exploratory nature of the analysis and the distribution of categories, no category collapsing was undertaken.

## 3. Results

### 3.1. Participant Characteristics (n = 245)

The final analytic sample comprised 245 practising orthotists from 28 countries. The largest country-level representation was from Jordan (18.8%), the United States (13.5%), Pakistan (11.8%), and India (10.6%). The full country distribution is presented in Table 1. The sample should be interpreted as a geographically diverse convenience sample rather than a globally representative cohort.

Most respondents held a Bachelor’s degree (67.8%), followed by a Master’s degree (17.1%), Diploma qualification (7.3%), and PhD (6.1%). In terms of clinical experience, respondents were distributed across all categories, with the largest proportion having 2–5 years of experience (33.5%), followed by those with more than 15 years (23.7%), while the remaining participants were distributed across the other experience levels.

Clinical exposure was high, with over half (53.1%) managing more than 50 AFO cases per year. Most respondents were involved across multiple stages of the AFO pathway, including fitting/delivery (97.6%), follow–up adjustments (94.7%), patient assessment (92.7%), and determining the technical design of the prescribed AFO (91.4%). Only 9.4% held formal legal prescribing authority, whereas 60% reported determining AFO design under physician referral.

Most respondents worked in public healthcare facilities (54.3%), followed by private clinics (31.4%) and university or academic institutions (13.5%), as illustrated in Table 1.

### 3.2. Awareness and Clinical Implementation of AFO–FC Tuning (n = 245)

**Figure 2:**
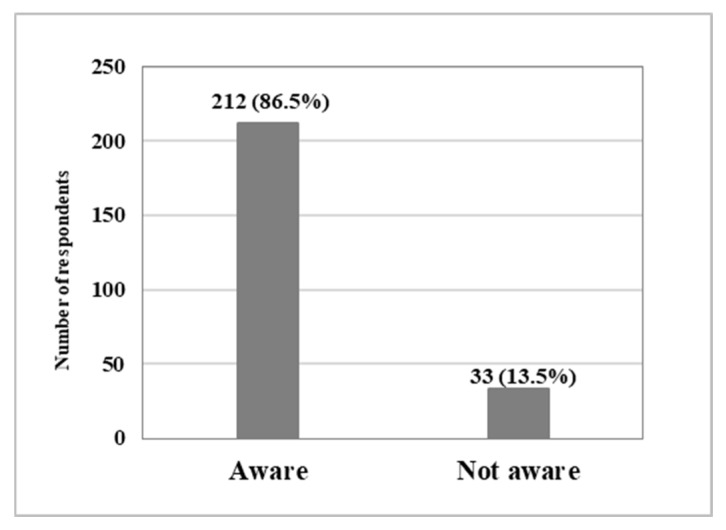
Self-reported awareness of AFO–FC tuning (*n* = 245).

Self-reported awareness and clinical implementation of AFO–FC tuning are summarised in Table 2 and illustrated in Figure 2.

Of the total sample (*n* = 245), 212 respondents (86.5%) reported awareness of AFO–FC tuning, and 176 (71.8%) indicated that they fully understood the concept. Despite this, routine implementation was lower, with 131 respondents (53.5%) reporting that they use AFO–FC tuning as standard practice for all patients prescribed an AFO.

**Figure 3:**
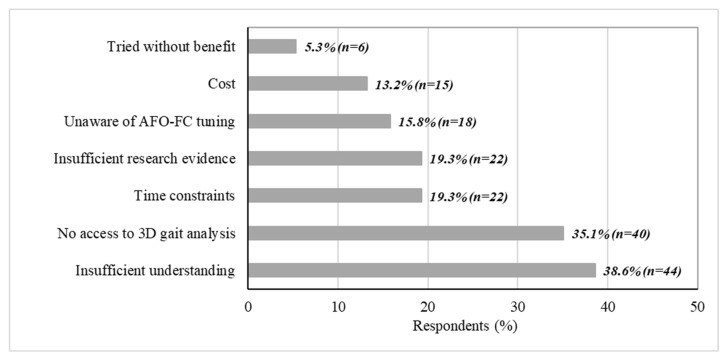
Reported barriers to routine implementation of AFO–FC tuning (*n* = 114).

Among respondents who did not routinely implement tuning (*n* = 114), the most frequently self-reported barriers were insufficient understanding (38.6%) and limited access to 3D gait analysis systems (35.1%). Additional barriers included time constraints (19.3%), perceived lack of high–quality research (19.3%), lack of awareness (15.8%) and cost considerations (13.2%). A small proportion (5.3%) reported having trialled AFO–FC tuning without observing clinical benefit, as illustrated in Figure 3.

### 3.3. Framework-Based Proficiency Classification Based on Biomechanical Framework (n = 212)

Framework-based proficiency was evaluated only among respondents who reported awareness of AFO–FC tuning (*n* = 212), as this subgroup completed the open-text items required for assessment using a predefined five-domain biomechanical framework.

#### 3.3.1. Distribution of Biomechanical Domains

Demonstration of individual biomechanical domains varied across responses. Patient-specific ankle range prescription consistent with R1a–R3 principles and footwear–orthosis interaction were the most frequently identified domains, whereas shank alignment during stance, measurement-based assessment, and proximal biomechanical effects were less commonly reported. The distribution of biomechanical domains is presented in Table 3.

#### 3.3.2. Cumulative Framework-Based Proficiency Classification

**Figure 4:**
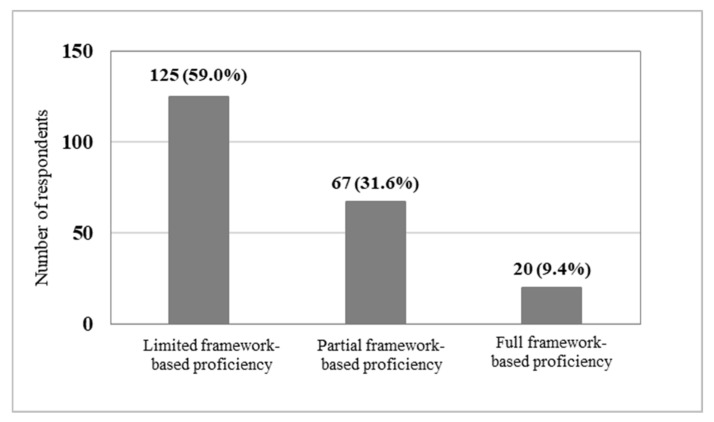
Distribution of framework-based proficiency levels based on cumulative domain scores (*n* = 212). Limited proficiency: 0–1 domains; partial proficiency: 2–3 domains; full proficiency: 4–5 domains.

Based on cumulative domain scoring (range 0–5), 125 respondents (59.0%) demonstrated limited framework-based proficiency (0–1 domains), 67 (31.6%) demonstrated partial framework-based proficiency (2–3 domains), and 20 (9.4%) demonstrated full framework-based proficiency (4–5 domains), as illustrated in Figure 4.

### 3.4. Associations with Framework-Based Proficiency Level

Associations between framework-based proficiency level (limited, partial, full) and selected professional characteristics (educational qualification, years of clinical experience, and annual number of AFO cases managed) were examined using chi-square tests of independence among respondents who reported awareness of AFO–FC tuning (*n* = 212).

No statistically significant associations were identified between framework-based proficiency level and years of clinical experience (χ^2^(8) = 9.12, *p* = 0.332), annual number of AFO cases managed (χ^2^(6) = 6.42, *p* = 0.378), or educational qualification (χ^2^(6) = 7.35, *p* = 0.290). Effect sizes were small across all comparisons (Cramér’s V = 0.08–0.15). Examination of expected cell counts indicated that several comparisons included low-frequency categories, particularly within higher qualification levels and the full-proficiency subgroup; therefore, these findings should be interpreted cautiously rather than as evidence of no association.

In contrast, a statistically significant association was observed between framework-based proficiency level and routine implementation of AFO–FC tuning (χ^2^(2) = 8.20, *p* = 0.017). Logistic regression analysis indicated that full framework-based proficiency was associated with higher odds of reporting routine implementation compared with limited framework-based proficiency (OR = 4.03, 95% CI 1.44–11.25, *p* = 0.008). However, this estimate should be interpreted with caution due to the small size of the full-proficiency subgroup (*n* = 20) and the relatively wide confidence interval, indicating limited precision. Partial framework-based proficiency was not significantly associated with implementation (OR = 1.20, 95% CI 0.63–2.28, *p* = 0.578).

## 4. Discussion

Although self-reported awareness of AFO–FC tuning was high, routine use was reported by only 53.5% of respondents, indicating a gap between familiarity with the concept and its consistent application in practice. Similar gaps between knowledge and implementation have been described across healthcare disciplines [33], where familiarity with a concept does not necessarily translate into structured clinical application. The proportion of respondents reporting insufficient understanding as a barrier further supports this interpretation, suggesting that awareness alone may be insufficient to ensure confident application of tuning principles in complex clinical contexts.

Among respondents who reported awareness, a subset met the criteria for full framework-based proficiency. Full framework-based proficiency was associated with reported routine use within this sample. This finding suggests an association between structured biomechanical reasoning and implementation rather than a causal relationship. Given the cross-sectional design and the framework-based measurement approach, it is not possible to determine whether higher framework-based proficiency facilitates implementation or whether clinicians who routinely implement AFO–FC tuning develop greater proficiency over time.

It is important to note that the classification reflects framework-based reasoning derived from structured coding of open-text responses, rather than a validated measure of clinical competence in real-world practice.

Notably, most respondents reported involvement across multiple stages of the AFO clinical pathway, including assessment, design specification, fitting, and follow-up. This suggests that variability in practice is unlikely to be explained solely by role limitations within the pathway and may instead reflect differences in how tuning principles are incorporated into clinical decision-making.

No statistically significant association was observed between framework-based proficiency level and educational qualification, years of clinical experience, or annual number of AFO cases managed. These findings should be interpreted with appropriate caution, particularly given variation in education systems, curricular content, and competency assessment, which may limit comparability across settings. The lack of association with educational qualification may reflect differences in how structured tuning frameworks are incorporated into training programmes. Similarly, experience and workload alone may not reflect how consistently biomechanical principles are applied in practice. Exposure to AFO provision does not necessarily translate into systematic application of tuning principles in routine care.

Overall, the findings suggest that awareness alone may not be sufficient to ensure routine use, whereas higher levels of framework-based proficiency were associated with reported routine implementation. This pattern may reflect an underlying association between awareness, framework-based proficiency, and implementation; however, the study design does not allow inference regarding progression or directionality between these constructs.

Experimental biomechanical studies have shown that structured AFO–FC tuning can modify lower-limb alignment, knee moment patterns, and spatiotemporal gait parameters in populations such as stroke and cerebral palsy [5,13,15,19,21,27]. However, these findings suggest that the availability of evidence and published clinical algorithms may not consistently translate into routine clinical use. Despite available guidance [8,12], only just over half of respondents reported systematic integration of tuning into practice. These findings suggest that access to evidence alone may be insufficient to ensure consistent clinical adoption, and that the ability to apply these principles within structured clinical reasoning may also play an important role.

Beyond AFO–FC tuning frameworks, variability in AFO design has been widely reported, including differences in material properties, stiffness, trimlines, and structural configuration [4,9,11,22,23,26]. These design characteristics influence lower-limb kinematics, joint loading, and energy expenditure during gait, indicating that device configuration interacts closely with alignment in determining functional performance [7,9,16,18]. Recent developments in patient-specific AFOs and advanced fabrication approaches further highlight the evolving role of design optimisation in orthotic management [10,22,23,25,27]. However, despite these advances, the integration of both design-related and alignment-related considerations within structured biomechanical reasoning remains inconsistent, and the relationship between AFO design, tuning strategies, and clinical implementation requires further investigation [12,13,28,29,30,31]. This may contribute to the variability in clinical practice observed in the present study and supports the role of structured approaches such as AFO–FC tuning in promoting consistent clinical decision-making.

The variability in implementation of AFO–FC tuning observed in this study is consistent with prior reports of inconsistent reporting and application of AFO–FC alignment parameters [28,29]. In particular, limited reporting of key parameters such as AA–AFO and SVA, as well as the extent to which these are individualised through tuning, has been identified. Such variability and lack of detail may hinder comparison across studies and may contribute to the inconsistency in clinical practice identified in the present study.

Reported barriers further clarify the translational gap identified in this study. Insufficient understanding of AFO–FC tuning principles and limited access to 3D gait analysis were the most frequently cited barriers. Although instrumented gait analysis may enhance measurement precision, previous research has shown that clinically observable parameters, such as shank-to-vertical angle, can be assessed with acceptable reliability using structured clinical measurement and video-based analysis [14,34]. This suggests that while advanced technologies such as 3D gait analysis may enhance precision, they are not a prerequisite for implementing structured AFO–FC tuning in routine clinical practice. Technological access alone may therefore not fully explain the observed variation in practice, and differences in clinical reasoning and confidence may also contribute. These observations are based on self-reported responses and reflect perceptions among non-routine implementers rather than objective barriers across all clinical settings.

The findings are consistent with previous work from the United Kingdom by Eddison et al. [31], who reported that approximately half of orthotists routinely incorporated AFO–FC tuning into practice. The comparable implementation rate observed in this sample suggests that inconsistent use may not be limited to a single healthcare context. However, interpretation should be cautious, as the sample was recruited through professional networks and online platforms, which may have introduced self-selection bias. Orthotists with a particular interest in AFO–FC tuning may have been more likely to participate, potentially inflating awareness and implementation estimates.

This study included respondents from 28 countries representing varied clinical contexts. The use of a predefined biomechanical framework with substantial inter-rater agreement strengthens methodological robustness. Combining self-reported measures with structured analysis of open-text responses allowed examination of both perceived and applied capability. However, several limitations should be considered. Recruitment through professional networks and social media may have introduced self-selection bias. In addition, routine implementation was self-reported and may be subject to recall bias and social desirability bias. The cross-sectional design prevents causal inference between framework-based proficiency and routine use. The five-domain framework represents a structured application of established biomechanical constructs rather than a validated psychometric tool. In addition, the English-only survey format may have influenced the depth and clarity of responses among non-native speakers, which may have affected proficiency classification independently of conceptual understanding. Open-text responses may also have been influenced by differences in response length, writing style, or survey fatigue, which may have affected the level of detail provided and, consequently, the assessment of framework-based proficiency. These limitations should be considered when interpreting the findings and may have influenced both the depth of responses and the resulting classification of framework-based proficiency. Country-level representation was uneven, and the findings should not be interpreted as nationally representative estimates of practice within individual countries.

The findings suggest that strengthening structured biomechanical reasoning may support more consistent integration of AFO–FC tuning into clinical practice. This may be particularly relevant within orthotist education and continuing professional development, where structured approaches to range-based ankle prescription (R1a–R3), shank alignment, footwear–orthosis interaction, and proximal biomechanical reasoning could contribute to greater consistency in clinical decision-making. Approaches such as guided supervision, case-based discussion, and reflective review of tuning practice may further support translation into routine care.

## 5. Conclusions

This study examined awareness, framework-based proficiency, and clinical use of AFO–FC tuning among practising orthotists. Although awareness was high, self-reported routine implementation was observed in just over half of respondents, indicating a gap between conceptual familiarity and consistent clinical application. Within this sample, higher levels of framework-based proficiency were associated with self-reported routine implementation; however, this association should be interpreted cautiously given the cross-sectional design, the self-reported nature of implementation, and the framework-based (non-validated) classification approach, which do not allow conclusions regarding causality or directionality. No statistically significant associations were detected between framework-based proficiency and educational level, clinical experience, or workload in this sample; however, these findings should also be interpreted cautiously given the sample size, category distribution, and limited statistical power for some comparisons. Overall, these findings should be considered indicative of a possible relationship that warrants further investigation in future studies using longitudinal or intervention-based designs.

## Figures and Tables

**Table 1:** Study sample characteristics (*n* = 245).

Characteristic	*n*	%
***Country of Practice***		
Jordan	46	18.8
United States	33	13.5
Pakistan	29	11.8
India	26	10.6
Saudi Arabia	14	5.7
United Kingdom	12	4.9
Iran	12	4.9
South Africa	9	3.7
Other countries (*n* = 20 countries) *	64	26.1
***Highest qualification***		
Diploma	18	7.3
Bachelor’s degree	166	67.8
Master’s degree	42	17.1
PhD	15	6.1
Other (bachelor–equivalent)	4	1.6
***Years of Clinical Experience as an Orthotist***		
<1 year	35	14.3
2–5 years	82	33.5
6–10 years	38	15.5
11–15 years	32	13.1
>15 years	58	23.7
***Number of AFO Cases Managed per Year***		
<10	21	8.6
10–30	48	19.6
30–50	46	18.8
>50	130	53.1
***Primary Workplace Setting***		
Public healthcare	133	54.3
Private clinic/hospital	77	31.4
University/academic	33	13.5
NGO/charity	2	0.8

* Includes Algeria, American Samoa, Australia, Belgium, Cameroon, Cyprus, Egypt, France, Ghana, Iraq, Ireland, Italy, Japan, Netherlands, Palestine, Portugal, Qatar, Sri Lanka, Syria, Turkey, and Yemen. Note: Percentages may not total 100 due to rounding.

**Table 2:** Self-reported Awareness and Clinical Implementation of AFO–FC Tuning (*n* = 245).

Outcome	Response	Frequency	Percentage
Self-reported awareness	Yes	212	86.5
	No	33	13.5
Self-reported full understanding	Yes	176	71.8
	No	69	28.2
Use AFO–FC tuning as standard practice for all patients	Yes	131	53.5
	No	114	46.5

**Table 3:** Distribution of biomechanical domains (*n* = 212).

Biomechanical Domain	Frequency	Percentage
Shank alignment during stance	52	24.5
Patient-specific ankle range prescription (R1a–R3)	88	41.5
Footwear–orthosis interaction	93	43.9
Measurement-based assessment methods	44	20.8
Recognition of proximal biomechanical effects	41	19.3

Note: Percentages do not sum to 100% because responses may be coded across multiple biomechanical domains.

## Data Availability

Data supporting the findings of this study are available from the corresponding author upon reasonable request.

## Supplementary Materials

The following supporting information can be downloaded at: https://www.mdpi.com/article/10.3390/jcm15082846/s1. Supplementary Materials S1: Full questionnaire, including open-text items used to assess framework-based proficiency in AFO–FC tuning.

## Abbreviations

The following abbreviations are used in this manuscript:

AFO	Ankle Foot Orthosis
AFO–FC	Ankle Foot Orthosis–Footwear Combination
AA–AFO	Ankle Angle in Ankle–Foot Orthosis
SVA	Shank–to–Vertical Angle
GRF	Ground Reaction Force
IRB	Institutional Review Board
CI	Confidence Interval
OR	Odds Ratio

## References

[1] Condie D.N., Hsu J., Michael J., Fisk J., Hsu J.D., Michael J.W., Fisk J.R. (2008). International Organization for Standardization (ISO) terminology. Atlas of Orthoses and Assistive Devices.

[2] Tyson S.F., Sadeghi-Demneh E., Nester C.J. (2013). A systematic review and meta-analysis of the effect of an ankle-foot orthosis on gait biomechanics after stroke. Clin. Rehabil..

[3] Choo Y.J., Chang M.C. (2021). Effectiveness of an ankle-foot orthosis on walking in patients with stroke: A systematic review and meta-analysis. Sci. Rep..

[4] Meadows C.B., Bowers R., Owen E., Hsu J.D., Michael J.W., Fisk J.R. (2008). Biomechanics of the hip, knee and ankle. Atlas of Orthoses and Assistive Devices.

[5] Carse B., Bowers R., Meadows C.B., Rowe P. (2015). The immediate effects of fitting and tuning solid ankle-foot orthoses in early stroke rehabilitation. Prosthet. Orthot. Int..

[6] Bowers R., Ross K. (2010). Development of a best practice statement on the use of ankle-foot orthoses following stroke in Scotland. Prosthet. Orthot. Int..

[7] Condie E., Bowers R., Hsu J.D., Michael J.W., Fisk J.R. (2008). Lower limb orthoses for persons with neuromuscular impairments. Atlas of Orthoses and Assistive Devices.

[8] Owen E.J. (2005). Proposed clinical algorithm for deciding the sagittal angle of the ankle in an ankle-foot orthosis–footwear combination. Gait Posture.

[9] Waterval N.F.J., Brehm M.A., Altmann V.C., Koopman F.S., den Boer J.J., Harlaar J., Nollet F. (2020). Stiffness-optimized ankle-foot orthoses improve walking energy cost compared to conventional orthoses in neuromuscular disorders: A prospective uncontrolled intervention study. IEEE Trans. Neural Syst. Rehabil. Eng..

[10] Kobayashi T., He Y., Wong C.L.Y., Koh M.W.P., Jor A., Orendurff M.S., Gao F. (2026). Impact of adjustable dorsiflexion range and stiffness in articulated ankle-foot orthosis on center of pressure progression in post-stroke gait. J. Biomech..

[11] Kerkum Y.L., Buizer A.I., van den Noort J.C., Becher J.G., Harlaar J., Brehm M.A. (2015). The effects of varying ankle-foot orthosis stiffness on gait in children with spastic cerebral palsy who walk with excessive knee flexion. PLoS ONE.

[12] Morris C., Bowers R., Ross K., Stevens P., Phillips D. (2011). Orthotic management of cerebral palsy: Recommendations from a consensus conference. Neurorehabilitation.

[13] Owen E.J. (2010). The importance of being earnest about shank and thigh kinematics, especially when using ankle-foot orthoses. Prosthet. Orthot. Int..

[14] Kerkum Y.L., Houdijk H., Brehm M.A., Buizer A.I., Kessels M.L., Sterk A., Noort J.C.v.D., Harlaar J. (2015). The shank-to-vertical angle as a parameter to evaluate tuning of ankle–foot orthoses. Gait Posture.

[15] Jagadamma K.C., Owen E., Coutts F.J., Herman J., Yirrell J., Mercer T.H., Van Der Linden M.L. (2010). The effects of tuning an ankle-foot orthosis–footwear combination on knee joint kinematics and kinetics in an adult with hemiplegia. Prosthet. Orthot. Int..

[16] Oudenhoven L.M., Kerkum Y.L., Buizer A.I., van der Krogt M.M. (2022). How does a systematic tuning protocol for ankle-foot orthosis–footwear combinations affect gait in children with cerebral palsy?. Disabil. Rehabil..

[17] Pongpipatpaiboon K., Mukaino M., Matsuda F., Ohtsuka K., Tanikawa H., Yamada J., Tsuchiyama K., Saitoh E. (2018). The impact of ankle-foot orthoses on toe clearance strategy in hemiparetic gait: A cross-sectional study. J. Neuroeng. Rehabil..

[18] Gatti M.A., Freixes O., Fernández S.A., Rivas M.E., Crespo M., Waldman S.V., Olmos L.E. (2012). Effects of ankle foot orthosis in stiff knee gait in adults with hemiplegia. J. Biomech..

[19] Choi H., Bjornson K., Fatone S., Steele K.M. (2016). Using musculoskeletal modeling to evaluate the effect of ankle–foot orthosis tuning on musculotendon dynamics: A case study. Disabil. Rehabil. Assist. Technol..

[20] Murphy D.P., Webster J.B., Lovegreen W., Simoncini A., Frontera W.R., DeLisa J.A., Braddom R.L. (2021). Lower limb orthoses. Braddom’s Physical Medicine and Rehabilitation.

[21] Condie D.N. (2008). The modern era of orthotics. Prosthet. Orthot. Int..

[22] Al Qaroot B., Sobuh M., Khanfar A., Al-Imyan F., Al-Laham E., Abd Razak N.A. (2025). Enhancing static alignment in hyperpronated feet using custom medial and lateral supports in rigid ankle-foot orthoses: A pilot study. Assist. Technol..

[23] Caravaggi P., Rogati G., Zamagni L., Boriani L., Arceri A., Ortolani M., Lullini G., Berti L., Leardini A. (2024). Functional evaluation of a novel fibreglass-reinforced polyamide custom dynamic ankle-foot orthosis for foot drop patients: A pilot study. Gait Posture.

[24] Van Duijnhoven E., Waterval N., Koopman F.S., Brehm M.-A. (2025). Optimizing gait with bidirectional tuning of ankle-foot orthosis stiffness in people with neuromuscular disorders: Preliminary results. Gait Posture.

[25] De Jong L.A.F., Kerkum Y.L., de Groot T., Vos-van der Hulst M., van Nes I.J.W., Keijsers N.L.W. (2021). Assessment of the shank-to-vertical angle while changing heel heights using a single inertial measurement unit in individuals with incomplete spinal cord injury wearing an ankle-foot orthosis. Sensors.

[26] Waterval N.F.J., Brehm M.A., Harlaar J., Nollet F. (2020). Description of orthotic properties and effect evaluation of ankle-foot orthoses in non-spastic calf muscle weakness. J. Rehabil. Med..

[27] Eddison N., Healy A., Needham R., Chockalingam N. (2020). The effect of tuning ankle-foot orthoses–footwear combinations on gait kinematics of children with cerebral palsy: A case series. Foot.

[28] Owen E., Hendy C.S. (2025). Do authors and editors comply with best practice reporting guidelines for ankle-foot orthosis interventions in studies involving children with cerebral palsy? A scoping review. J. Prosthet. Orthot..

[29] Ridgewell E., Dobson F., Bach T., Baker R. (2010). A systematic review to determine best practice reporting guidelines for ankle-foot orthosis interventions in studies involving children with cerebral palsy. Prosthet. Orthot. Int..

[30] Goihl T., Rusaw D.F., Roeleveld K., Brændvik S.M. (2024). Provision of ankle-foot orthoses for children with cerebral palsy in Norway. Disabil. Rehabil. Assist. Technol..

[31] Eddison N., Chockalingam N., Osborne S. (2015). Ankle-foot orthosis–footwear combination tuning: An investigation into common clinical practice in the United Kingdom. Prosthet. Orthot. Int..

[32] World Medical Association (2013). World Medical Association Declaration of Helsinki: Ethical principles for medical research involving human subjects. JAMA.

[33] Graham I.D., Logan J., Harrison M.B., Straus S.E., Tetroe J., Caswell W., Robinson N. (2006). Lost in knowledge translation: Time for a map?. J. Contin. Educ. Health Prof..

[34] Nguyen B.T., Baicoianu N.A., Howell D., Peters K., Steele K.M. (2020). Accuracy and repeatability of smartphone sensors for measuring shank-to-vertical angle. Prosthet. Orthot. Int..

