# Supplementary material for: Awareness, Framework-Based Proficiency, and Clinical Implementation of Ankle Foot Orthosis–Footwear Combination (AFO–FC) Tuning: A Cross-Sectional Survey
Source: J Clin Med. 2026 Apr 9;15(8):2846. doi: 10.3390/jcm15082846 (PMC13115975; doi:10.3390/jcm15082846)
Supplement: Supplementary file 1 [file jcm-15-02846-s001.zip › jcm-4211165-supplementary.pdf]

Note: The questionnaire comprised three sections. Section 2 items (7–13) were adapted from the questionnaire developed by Eddison et al. [31], with core wording retained to support comparability. Section 3 contains the open-text items used for framework-based proficiency assessment and was completed only by respondents who reported awareness of AFO–FC tuning (answered "Yes" to Question 7).

Question	Response
**Section 1. Demographic Information**	
1. Do you work as an Orthotist?	☐Yes<br>☐No
2. What is the highest level of education you have completed?	Diploma<br>Bachelor's degree<br>Master's degree<br>PhD<br>Other (please specify):
3. How many years of clinical experience do you have as an orthotist?	Less than 1 year<br>2–5 years<br>6–10 years<br>11–15 years<br>More than 15 years
4. Approximately how many AFO cases do you manage per year (assessment, fitting, follow-up, and/or adjustments)?	Less than 10<br>10–30<br>30–50<br>More than 50
4.1 In your setting, what is your role in the AFO pathway? (Select all that apply)	Assess/evaluate patients for AFOs<br>Determine the technical design parameters of the prescribed AFO (e.g., trimlines, stiffness, ankle angle, alignment)<br>Fabricate/manufacture AFOs<br>Fit/deliver AFOs<br>Provide follow-up adjustments/tuning<br>Formally prescribe AFOs (legal prescriber)<br>Informally prescribe: generally under physician referral
5. In which country do you currently practise?	
6. What institution do you work for?	Public (government-funded) healthcare facility<br>Private hospital or private clinic<br>University or academic institution<br>Non-governmental organisation (NGO) or charity<br>Military medical service<br>Other (please specify)
**Section 2. Awareness, Implementation, and Clinical Practice (Adapted from Eddison et al. [31])**	
7. Are you aware of AFO-FC tuning?	☐Yes<br>☐No
8. Do you fully understand AFO-FC tuning?	☐Yes<br>☐No

9. Do you use AFO-FC tuning as standard practice for all patients who are prescribed with an AFO?	☐Yes<br>☐No
9.1 If no, what is preventing you from using AFO-FC tuning? (Multiple selection allowed)	I don't fully understand it<br>I don't have access to 3D gait analysis<br>It's too time-consuming<br>It's too costly<br>I'm unaware of AFO-FC tuning<br>There's not enough quality research<br>Tried it but didn't see any benefit
10. How do you decide which patients will benefit from AFO-FC tuning?	I have set criteria<br>I tune all patients who are prescribed with an AFO<br>It depends whether I have enough time
10.1 If you have set criteria, please describe them: (Open-ended)	
11. Do you use 3D gait analysis to tune AFO–FCs?	☐Yes<br>☐No
11.1 Do you use video analysis?	☐Yes<br>☐No
11.2 Do you tune by eye alone?	☐Yes<br>☐No
11.3 Do you use any other method?	☐Yes<br>☐No
12. Do you take AFO design into consideration when deciding whether to tune an AFO-FC?	☐Yes<br>☐No
12.1 If yes, please state the design criteria which would prevent you from tuning the AFO-FC: (Open-ended)	
13. Do you take physical ability of the patient into account when deciding whether the AFO-FC should be tuned?	☐Yes<br>☐No
13.1 If yes, please state physical criteria which would prevent you from tuning an AFO-FC: (Open-ended)	

SECTION 3: Clinical Reasoning and Application of AFO–FC Tuning
(To be completed only by participants who answered “Yes” to Question 7)

14. How do you determine the ankle angle of a rigid AFO? Please describe any range of motion assessments, alignment targets, or clinical measurements used? (Open-ended)	
15. When adjusting an AFO–footwear combination after fitting, what biomechanical factors guide your modifications? (Open-ended)	
16. During stance, how do you determine whether the AFO–footwear combination is appropriately positioned? (Open-ended)	
17. Do footwear characteristics influence your tuning decisions?	☐Yes<br>☐No
17.1 If yes, please describe how footwear influences alignment or function. (Open-ended)	
	☐Yes

18. Do you use any measurement-based methods to assess alignment during tuning?	☐No
18.1 If yes, please specify: (Open-ended)	
19. What biomechanical effects at the knee or hip do you expect when AFO–FC tuning is successfully achieved? (Open-ended)	
